# Understanding, beliefs and perspectives of Aboriginal people in Western Australia about cancer and its impact on access to cancer services

**DOI:** 10.1186/1472-6963-9-132

**Published:** 2009-07-31

**Authors:** Shaouli Shahid, Lizzie Finn, Dawn Bessarab, Sandra C Thompson

**Affiliations:** 1 Centre for International Health, Curtin University of Technology, GPO Box U1987, Perth WA 6845, Australia

## Abstract

**Background:**

Despite a lower overall incidence, Aboriginal Australians experience poorer outcomes from cancer compared with the non-Aboriginal population as manifested by higher mortality and lower 5-year survival rates. Lower participation in screening, later diagnosis of cancer, poor continuity of care, and poorer compliance with treatment are known factors contributing to this poor outcome. Nevertheless, many deficits remain in understanding the underlying reasons, with the recommendation of further exploration of Aboriginal beliefs and perceptions of cancer to help understand their care-seeking behavior. This could assist with planning and delivery of more effective interventions and better services for the Aboriginal population. This research explored Western Australian (WA) Aboriginal peoples' perceptions, beliefs and understanding of cancer.

**Methods:**

A total of 37 Aboriginal people from various geographical areas within WA with a direct or indirect experience of cancer were interviewed between March 2006 and September 2007. Interviews were audio-recorded, transcribed verbatim and coded independently by two researchers. NVivo7 software was used to assist data management and analysis. A social constructionist framework provided a theoretical basis for analysis. Interpretation occurred within the research team with member checking and the involvement of an Aboriginal Reference Group assisting with ensuring validity and reliability.

**Results:**

Outcomes indicated that misunderstanding, fear of death, fatalism, shame, preference for traditional healing, beliefs such as cancer is contagious and other spiritual issues affected their decisions around accessing services. These findings provide important information for health providers who are involved in cancer-related service delivery.

**Conclusion:**

These underlying beliefs must be specifically addressed to develop appropriate educational, screening and treatment approaches including models of care and support that facilitate better engagement of Indigenous people. Models of care and support that are more culturally-friendly, where health professionals take account of both Indigenous and Western beliefs about health and the relationship between these, and which engage and include Indigenous people need to be developed. Cultural security, removing system barriers and technical/scientific excellence are all important to ensure Indigenous people utilise healthcare to realise the benefits of modern cancer treatments.

## Background

A series of reviews recently highlighted differences in the epidemiology and the poorer outcomes of cancer in Indigenous people in Africa, Polynesia and Australia [1-4]. Given the complexity, expense and technology involved in modern cancer treatment, such disparities in cancer outcomes are unsurprising in the developing world. However, in Australia and New Zealand, the differences in cancer survival for Indigenous compared to the non-Indigenous populations warrants further investigation as these countries have well developed health systems offering universal healthcare for their citizens. Aboriginal and Torres Strait Islanders are the original inhabitants of Australia, and often referred to as Indigenous Australians. In this paper, the term Indigenous has been used to refer to first nation or the original inhabitants prior to colonisation in different countries including Australia. However, Aboriginal is the term preferred by vast majority Indigenous people of WA and is used for study participants.

Indigenous Australians have a lower incidence of cancer overall than the non-Indigenous population[3,5] although the epidemiology differs and includes higher rates of cancers with a poor prognosis. The improvement of around 20% in cancer survival in Australia over the last twenty years[6] has not been shared by Indigenous survival figures, with Indigenous Australians 2.5 times more likely to die within five years of cancer diagnosis[7]. The factors underlying these poorer outcomes include health and social disadvantage, health risk behaviours, lower participation in screening programs, later diagnosis of cancer, lower uptake and poorer compliance with treatment and poorer continuity of care[3,8-11]. While the relationship between knowledge, attitudes, beliefs and behaviours is complex, most theories of behaviour acknowledge that beliefs and attitudes have also an important influence upon an individual's decision to access healthcare.

Health, health practices and care-seeking behaviour are culturally bound[12]. Culture is enmeshed in historical, social, economic and political relationships and processes[13] and influences the ways that people understand cancer which, in turn, affects their decision-making around care-seeking and accessing of services [14-18]. Beliefs such as 'talking about something can cause it to happen',[18] screening is unnecessary in the absence of symptoms; relating cancer with black magic; and religious beliefs about destiny have been found to impede early detection and treatment[12,19,20].

The system of health care provision often fails to meet the needs of vulnerable groups. In Australia, Indigenous people are particularly at risk because, on a range of health and social indicators, they are the most marginalised of any identifiable group. While individual disadvantage is not unique to Indigenous people, it is the coalescence of markers of disadvantage and the resulting health outcomes that make understanding Indigenous beliefs particularly important. Cunningham et al recommended that messages from qualitative studies exploring the views and understanding Indigenous people with cancer must be taken into account[3].

Considerable differences exist in the perception and definition of health, healthy living, wellbeing, illness, and the meaning of disease and death between Indigenous Australians and the dominant Anglo-Australian society [21-23]. Few attempts have been made to systematically explore Indigenous views about cancer[24]. This paper reports the first comprehensive Australian study of Aboriginal beliefs about cancer.

## Methods

### Ethics approval

The research adhered to guidelines for ethical conduct of Aboriginal and Torres Strait Islander health research[25], and was approved by the Human Research Ethics Committee (HREC) of Curtin University, the Western Australian Aboriginal Health Information and Ethics Committee, and the ethics committees of the Royal Perth and Sir Charles Gairdner Hospitals. Approval was also obtained from local Aboriginal Health Services. Efforts were made throughout to conduct the study in ways that would build capacity and help equalize power between Aboriginal participants and researchers[26,27]. An Aboriginal Reference Group (ARG) provided input throughout.

### Data collection

This was a qualitative study in which the 'meaning of cancer' was explored among Aboriginal people in WA. Participants were Aboriginal males and females who were cancer patients/survivors (n = 14), family members of people with or who had died from cancer (n = 16) and health service providers (n = 7) (Table 1). All spoke English and in-depth interviews between March 2006 and September 2007 explored participants' beliefs and how they felt about and made sense of cancer. A semi-structured interview schedule guided the interviews, with participants encouraged to introduce topics of importance to them. Data collection continued until there was repetition of themes.

**Table 1:** Characteristics of study participants

**Aboriginal Participants (n = 37)**	
**Area of Residence**	
Urban participants	15
Regional participants	22

**Category of Respondent**	
Patients	14
Family Members	16
Health Service Providers	07

**Sex**	
Male	8
Female	29

**Age (years)**	
30–39	5
40–49	19
50–59	9
60+	4

### Data analysis

The social constructionist framework which emphasizes the complex development and interaction between knowledge, meaning, interpretation and power in the constitution of belief systems[28] assisted understanding in how the cultural meaning of cancer impacted upon participants' care-seeking behaviour. Social constructionists hold assumptions that individuals develop subjective meanings of their experiences that are guided, to some extent, by their beliefs and understanding which are constructed and negotiated socially and historically[29].

QSR NVivo7 software was used to manage data and support analysis. Thematic analysis of participants' transcribed interviews involved open coding independently by two researchers. Participants' responses were broken down into distinct units of meaning, or codes. Member checking was used to clarify whether emerging themes were an accurate reflection of the participants' experiences. The axial coding stage involved continuous comparisons of codes with one another to discover links between the categories[30], with related categories combined and compared to new data, arranged and rearranged to identify the key themes. To maximize reflexivity and rigour, all stages were discussed within the research team for verification and clarification of emerging themes[31]. Interpretation was assisted by consultation with ARG members and through presentations and feedback at various Aboriginal group meetings.

## Findings

A range of beliefs were reported, some by most participants while others occurred less commonly. Beliefs foreign to the western scientific paradigm were just as likely to be expressed by urban and educated residents, including those who had worked within mainstream health settings. In reporting, emphasis is given to findings at odds with western medicine or experiences common to many participants.

### Perspectives and understanding of cancer

#### Spirituality and cancer

Some participants associated cancer with the spiritual world of curses, a form of punishment resulting from some misdeed the person had done in the past. Blaming others or a particular life experience as a cause of sickness is widespread within Aboriginal communities where spirituality exerts a powerful influence upon the notion of wellbeing[32,33]. Such attribution of cancer to spiritual causes can lead to fatalism, acceptance of the disease without question and not seeking help for it – *"Aboriginal people have this notion of being sung... it's basically a bad magic put on somebody." *As a consequence of such beliefs, people may feel ashamed about their *"wrong-doing" *and hide their symptoms from others, delaying diagnosis or not pursuing treatment [6]. As stated by one family member:

"...it was almost like you deserved it or there was definitely this sense of shame. It was whispered. If someone died of a heart attack you would say that, but... all this cancer stuff was a whispered sort of stuff."

Relating cancer to spiritual causes is a pre-Enlightenment phenomenon and continues in Indigenous people elsewhere[19,34] and in other cultures[18,35]. It can often work as a coping mechanism to help overcome loss. One participant who had trained as a nurse in telling the story of her daughter's death from cancer talked of her daughter embodying her grandmother's spirit, being sent to explore her grandmother's country and ancestors. The daughter's death was accepted as inevitable, an outcome whispered to her by her mother's spirit long before her daughter's death.

#### Fatalism and cancer as a death sentence

Participants expressed deep fear and fatalistic expectations about cancer: '*cancer equals death*'. This belief was considered as a major factor explaining why people ignore early symptoms and do not access treatment even after medical diagnosis.

"... they are just scared, because at the very end they know they are going to die. As soon as they hear the word cancer they are scared. Cancer is a scary word in the Aboriginal communities."

Fear of cancer is universal, yet attitudes have changed in most developed countries where messages emphasizing early detection and cure are publicised. Traditional attitudes towards cancer involving hopelessness and death have been replaced by a culture of hope [16], and the belief that cancer is incurable has been largely overcome[36]. However, the pessimistic attitudes towards cancer in this study reinforce similar findings in other Indigenous peoples[19,34,37], with their unfortunate life-threatening consequences [19]. Participants considered that Indigenous interpretations of cancer as a 'death sentence' reflected the outcomes they have seen.

*"It's sort of like your world crumbles. All we know about cancer is you die from cancer, not so much that cancer can be cured. You always know that as soon as you get cancer you are gone..., you are a goner"*.

Few members of their families and communities were seen to survive cancer:

"I saw my Mum goes through chemo and radiation... I saw my baby brother go through it. I seen my first cousin goes through it, and all my aunties all had cancer, all my mum's sisters. They have all passed away with cancer...."

Spirituality, fatalism and religion all co-existed. Eight participants said that contracting cancer was beyond the control of an individual, many believing that one was chosen by God to get it.

"I don't think that it's something you can prevent, it's just people are chosen. ... you can go and have tests every six months, and one day you could just have it and it's been there the whole time..."

Many cultures hold similar beliefs regarding destiny and God's will[18]. Such fatalistic beliefs are strongly associated with delays accessing pap smears and follow-up of abnormalities[17]. Patients with a fatalistic outlook are less likely to take steps to lower their cancer risk[38], and accept their "imminent demise and refuse potentially life-saving treatment"[16].

Passivity existed alongside fatalism, expressed as belief that nothing could prevent a person from getting cancer: "*When your time's up, your time is up, and you cannot do anything about it"*. One participant emphasized latent internal causes – *'everybody has got cancer cells in their body, but it just takes something to spark it off'*. The participant was not referring to spontaneous cell abnormalities escaping normal immune surveillance[39] but rather elaborated on *"something" *as anything starting from curse, bad spirit, stress and bad luck, very different from the attribution in scientific explanations. Furthermore, such views do not acknowledge health behaviors as known risk factors for cancer[2]. Some participants voiced not wanting to worry about any sickness until they faced it, wanting to continue to live the way they had despite awareness of the associated risks:

*"...you shouldn't stop your life because of all these sicknesses... that's just something that happens, and you deal with it when it comes along... so until then ... just forgetting... laughing..."*.

#### Unrealistic expectations of treatment

Contradicting the view 'cancer means death' were comments that Indigenous people accessing cancer treatments often put too much faith in doctors, believing they could fix their health problem. One respondent referred to "*the doctors as gods ...they are the ones that are going to fix it, the miracle-makers"*. This confidence existed without understanding the complexities of cancer staging, co-morbid physical conditions, treatment options and the prognosis of different types of cancer. After finishing treatment, some thought they had been cured, that the cancer had gone and they could get on with their life normally, perhaps not attending for follow-up check-ups. One cancer patient spoke about her mother who believed she was cured by a mastectomy:

"she keeps saying, 'I have no more cancer... oh they took it all now...' And I keep saying to her, 'Mum, no, it's not true. It's still in your body. Although they took your bubies off, you still got the disease. You got to be careful...."

Both cancer deaths and recurrences led to disappointment, often considered as an over-reliance or misplaced trust in doctors and western medicine. This could strengthen the distrust Indigenous people commonly feel towards western organisations including doctors and the medical system[40,41]. Personal stories of an individual's disillusionment with the medical system spread in the community, in turn influencing the choices others make around screening, early presentation and treatment for cancer. Distrust and negative experiences in the health system have similarly adversely impacted cancer care-seeking of other minorities[42].

#### Cancer is contagious

While not universal, some Aboriginal people believe cancer is contagious. Participants spoke about feeling isolated after diagnosis by the distancing behavior of some friends, family members and others who believed they were at risk of catching the cancer.

"There was a couple who were really scared of but there was one lady... she actually couldn't sit next to me. She sat across the room from me. She wouldn't talk to me for a long time, because she was scared...

Other studies have shown strong links between a person's beliefs about contagiousness, hiding their sickness and avoiding treatment, and feeling stigmatized or fearing being ostracized[19,29]. Although the belief that cancer is contagious is almost non-existent among the general population in WA[36], it can persist among some people from diverse cultural backgrounds[43].

#### Understanding of cancer

Fatalistic beliefs and attitudes in the general population have changed as a result of scientific research, dissemination of information and education to help people understand the biological basis of cancer and modern treatment. However, the poorer educational background and socio-economic conditions of many Indigenous Australians have limited their access to information and understanding about disease. A lack of knowledge about types of cancer, symptoms, treatment options and outcomes was apparent, with some respondents having never considered what type of cancer a loved one had. Irrespective of geographic residency, respondents reported not initially recognising the cancer symptoms and delaying getting them checked. One woman had never taken the time to find out about serious illnesses and *"didn't have a clue that it was the start of ...where that brown part puckering up, tightens up." *The idea of self examination, of checking yourself for abnormalities that appeared to be foreign: *"He asked how long I had the lumps (under my arms and neck)...I asked what lumps, I hadn't even felt any lump."*

Attribution of cause for cancer was often unsophisticated:

"She thought her nose was bleeding because her husband punched her in the nose, and I don't know that she ever understood that it was anything more than that, because that was her experience was, everything was all right until he punched her in the nose and it started bleeding."

Close family members were often unsure about what was happening to relatives and felt they could have helped more had they been better informed or more knowledgeable. Comments such as "*we didn't know what was happening" *and *"We didn't know that she got cancer until she died" *were common. These comments reflect communication problems for Aboriginal people within health facilities, and ignorance about cancer symptoms such as weight loss, anorexia and bleeding. *"I didn't relate dad's condition to cancer. I found out later when I read up about it ...it was... almost ten years after I lost my dad."*

Working in health services had improved some participants' understanding and knowledge about cancer but they commented on the lack of understanding in the Indigenous community: *"a lot of Indigenous people...I suppose 70 to 80 per cent, wouldn't really know properly." *Another commented:

"I don't think they understand it. They don't understand about prevention. They don't understand about early detection and screening. Really, I felt that – from working there – some of their experiences or their understanding is so simple, it is very childlike."

Poor knowledge about cancer warning signs, screening and risk factors among minority populations have been reported elsewhere[17,44,45]. This limited understanding contributes to the many communication gaps between practitioners and patients, increasing patients' frustration with doctors and the medical system.

#### Perceptions of cancer screening

Understanding of cancer screening, its purpose and importance was often limited and vague. Ambivalence about participation in screening is unsurprising, particularly if there is a fatalistic view of cancer. As one health worker commented, *"It's a sense that why I am doing a pap smear is to tell them they have got cancer and they are going to die from it...." *Some participants believed that accessing screening would prevent cancers from occurring, with a few viewing screening as an early diagnostic tool. The discomfort and inconvenience of screening, "*fear of knowing*", "*fear of having their breast squashed*" and the "*shame*" of being touched by another person, were relayed as factors why Aboriginal women do not participate in screening programs. One woman referred to the shame of letting another woman touch her breasts or private parts and concern of being stigmatized as "*lesbian*". Prohibitions were also in place for men.

"The prostate thing with the Aboriginal men is... like I say is a 'taboo'... area. They will not go and get a simple test done by the doctor... they feel very funny about it, and so they usually leave it until the last minute, and sometimes that's just too late."

#### Urban versus rural/remote differences

The research explicitly proposed to examine differences in beliefs and understandings of regional compared to urban Aboriginal people who were considered likely to have more acculturation to western understandings. However, the range of views and beliefs did not map readily on the basis of geography or residence. Aboriginal people are mobile and many participants maintain connection with their homeland and culture despite living elsewhere[32,46]. One participant commented about returning to her country:

"I just feel replenished. My soul is just ... sort of filled up again. I'm home; 'This is where I feel so good.' And it feels good in here. I might not feel healthy. I might have a cold or whatever, but inside I feel ...It just fills me up. It's like a warm bath inside..."

#### Bush medicine and traditional Aboriginal healing practices

Other than a few participants *"never brought up that traditional way"*, use of bush medicines for cancer was widely reported. The Indigenous "holistic view" of life [47,48] in which health is defined as their total wellbeing [49] was frequently iterated: *"Healing is not just physical; it's mental, emotional and spiritual as well." *For Indigenous people, *"a positive outlook and in-look" *was considered necessary to be healthy:*"If you feel good inside regardless of your health, it will help you in any medical problems"*. The majority of cancer patients had used bush medicine either sequentially or concurrently[50] with Western medicines.

"There is something in it...that is good for your insides, just as a cleanser. Makes all your body organs healthy and strong, it gets rid of all your internal stress."

Participants also emphasized the importance of cancer being diagnosed at an early stage for bush medicine to work. Even when cure was not possible, bush medicine was used for palliation, often signifying a re-connection to land, ancestral and spiritual roots that enhanced the person's overall wellbeing.

Use of complementary and alternative medicine is high among cancer patients[51,52]. Certain populations, including Indigenous people worldwide, have their own approaches to healing as part of their culture [50,53]. Western health practitioners need to understand and acknowledge traditional healing and treatment approaches in order to work and communicate effectively with Indigenous patients.

## Discussion

**Figure 1:**
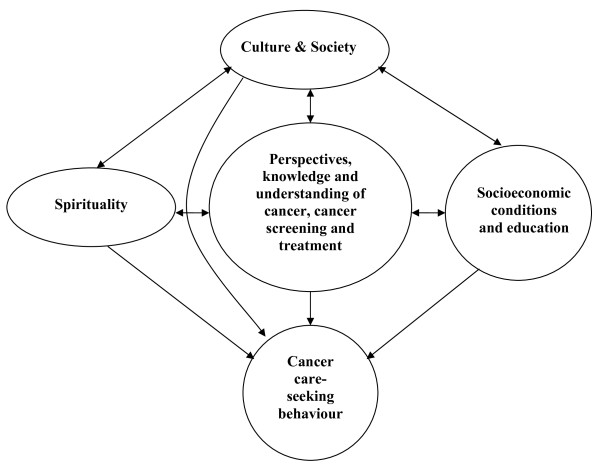
**Meaning-making around cancer and its impact on care-seeking behaviour**.

Patients' beliefs influence their care-seeking behaviour for cancer-related services. Considerable literature shows that Indigenous people are often unwilling to use mainstream health services[32], and cancer services are no exception, with lower Indigenous uptake and compliance with cancer treatments. To increase Indigenous people's willingness to accept modern oncology treatments will require a different approach to engaging them in treatment, one which understands and addresses their concerns and provides more psychosocial and holistic care alongside Western medical treatment. Yet to date, understanding Indigenous psycho-social and cultural beliefs and fears about cancer and their impact on care-seeking has been largely neglected despite qualitative methodology being useful for health services research in multicultural settings[54]. The findings from this study align with the social constructionist approach [55], which considers how culture, social life, social interactions and relations shape people's beliefs and understanding about cancer which in turn influences their cancer care-seeking behaviour. This relationship is illustrated in Figure 1.

Beliefs, understanding and interpretation stem from shared values, social relations within and between the family and the community, past experiences, cultural identity, and values[56,57]. Like many other cultures[18,58], notions of 'living well', 'sickness' or 'illness' present a complex, dynamic picture in Indigenous populations. Concept of health and wellbeing range from 'absence of symptoms of illness' to a more complex and holistic view of health as the consequence of physical, environmental, mental, and spiritual balance. Lifestyle factors, a person's social relationship with others and harmony with their culture are considered interrelated influences on health[59]. Thus, there is poor compatibility between the underlying principles of the Western reductionist medical system and traditional Indigenous health beliefs[50,59], or even those more recently socially constructed, which shape their care-seeking and willingness to engage with services. Changing community narratives by respecting Aboriginal culture, through education and improving life circumstances and trust of the health system will be important to change the constructs within which cancer beliefs are framed. Health care providers must consider, respect and respond to these needs if they are truly committed to improving Indigenous health outcomes.

The overall pessimistic attitude of Aboriginal people towards cancer as a 'killer' resonates with that of many other cultures [60,61]. Participants' understanding that cancer can often be delayed or overcome with timely Western medical treatment was limited. They had limited access to relevant information, and the shame attributed to cancer and reluctance to talk about it meant stories of survival were not widely disseminated. Appropriately targeted education campaigns, Aboriginal cancer support services and opportunities for Aboriginal survivors of cancer to be advocates in their communities are needed.

Traditional beliefs are not simply displaced by western biomedical understanding, they can co-exist. A study of Indigenous Australian and Papua New Guinean health science students showed that the Indigenous university students conceptualized health and illness in a way which accommodated biomedical science within an integrated scheme of mental, physical and spiritual well-being[59]. Education in western health care did not alter their core philosophy towards life and their spiritual belief system. Therefore, ensuring services are culturally appropriate is important for all Indigenous people, including those with higher levels of education and living in urban areas.

Many Aboriginal people retain a preference for using bush medicine and traditional healing, even for a "western" disease like cancer. The "doctor-dependent, hospital-based, curative western health care model"[58] for treating cancer does not generally recognize, and incorporate traditional systems for healing. However, the combined use of both types of expertise can optimise the response to various health problems, including cancer.

Participants argued for cross-cultural educational initiatives where western cancer support entails an understanding, acknowledgement and acceptance of Aboriginal belief systems and that they are different to western understandings. Aboriginal people also need to understand that western understandings are different. Consideration of cultural differences is essential for health care providers to fully appreciate the impact of this disease on patients' physical and mental well-being. Mutually appreciative understanding of cultural differences is a key to encouraging Indigenous people's willingness to participate in health care to enhance early detection, develop appropriate interventions and ultimately improve cancer outcomes[62].

Paramount to cancer being curable is diagnosis at a stage before spread, when treatments are most effective. The association of cancer with death reflects the tragic reality that Indigenous people are often diagnosed with cancer at an advanced stage, and consequently die within a short period of diagnosis [5,63,64]. Participants commented that some people who develop symptoms consistent with cancer avoid assessment and diagnosis, preferring to hide their symptoms, again a phenomenon not restricted to Aboriginal people[65]. In this way, they avoid confronting their diagnosis and potential mortality for a period of time. This situation needs to be approached through effective education about risks, symptoms and treatments for cancer. In addition, reducing barriers in access, providing more culturally secure health service provision, increasing the visibility of Aboriginal cancer survivorship and focusing attention on the importance of early diagnosis are strategies that can enhance cancer mortality in Indigenous communities.

The study was undertaken only in Western Australia, and it may not reflect the views of Aboriginal people throughout Australia who have different cultural traditions and beliefs. Men were under-represented in our participants which may be due to the primary interviewer being female, the predominance of women among the ARG members and in the community-based health workforce, and the higher utilisation of health services by women compared to men[66,67]. Another limitation was that participants needed to be able to speak English, and this proficiency in English would undoubtedly have some affects on acculturation and exposure to western understandings of health and illness.

## Conclusion

**Figure 2:**
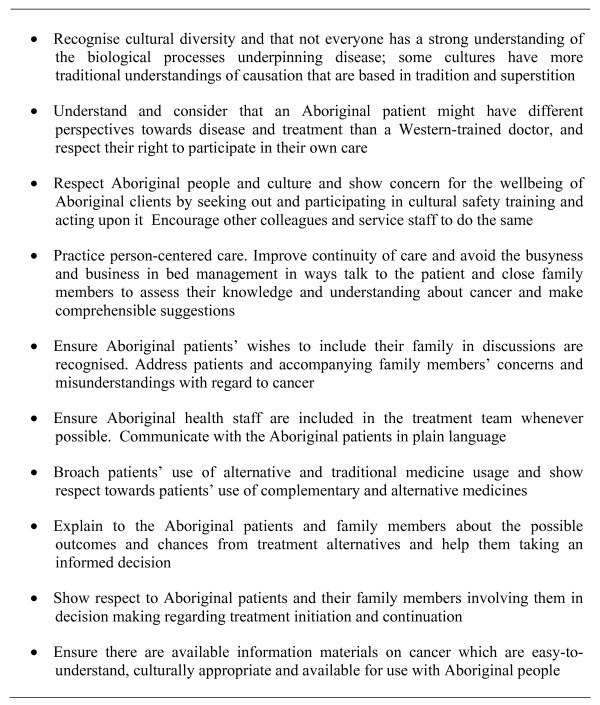
**Lessons for health practitioners**.

Beliefs are important but are only one influence on health behaviour. The concept of cultural safety requires a change in emphasis, away from the failings of individual patients (to attend, to comply etc) to critical examination of system factors in health care delivery that may interfere with an individuals' or a collectivity's willingness to attend a health service or take up treatments that are available. In addition to clinicians needing an understanding of cultural beliefs, a focus on the practice, skills and behaviour of the health system is required so that it appropriately responds to barriers and incorporates "culture" into service delivery'[68]. Models of care and support that are more culturally-friendly, where health professionals take account of both Indigenous and Western beliefs about health and the relationship between these, and which engage and include Indigenous people need to be developed[69]. Key lessons for health practitioners are summarised in Figure 2. Only by combining cultural security with technical/scientific excellence and removing system barriers will the potential benefits of modern treatments be realised through increased willingness and ability of Indigenous people to access and participate in healthcare.

## Competing interests

The authors declare that they have no competing interests.

## Authors' contributions

SS participated in the project's design, carried out the data collection and analysis for this project, prepared the initial draft. LF was involved in the data analysis phase and writing. DB helped interpret findings, and commented upon drafts of the manuscript. SCT coordinated the whole project, participated in the design and assisted with the conduct of the study and writing. All authors read and approved the final manuscript.

## Pre-publication history

The pre-publication history for this paper can be accessed here:


